# The Omics Landscape of Long COVID—A Comprehensive Systematic Review to Advance Biomarker, Target and Drug Discovery

**DOI:** 10.1111/all.16526

**Published:** 2025-03-14

**Authors:** Nadia Baalbaki, Elise M. A. Slob, Samuel W. Kazer, Mahmoud I Abdel‐Aziz, Harm Jan Bogaard, Korneliusz Golebski, Anke H. Maitland‐van der Zee

**Affiliations:** ^1^ Department of Pulmonary Medicine Amsterdam UMC Amsterdam the Netherlands; ^2^ Amsterdam Institute for Infection and Immunity Amsterdam the Netherlands; ^3^ Amsterdam Public Health Amsterdam the Netherlands; ^4^ Department of Clinical Pharmacy Haaglanden Medical Centre The Hague the Netherlands; ^5^ Department of Clinical Pharmacy and Toxicology Leiden University Medical Center Leiden the Netherlands; ^6^ Division of Gastroenterology, Hepatology, and Nutrition Boston Children's Hospital Boston Massachusetts USA; ^7^ Department of Immunology Blavatnik Institute, Harvard Medical School Boston Massachusetts USA; ^8^ Broad Institute of MIT and Harvard Cambridge Massachusetts USA; ^9^ Ragon Institute of MGH, MIT and Harvard Cambridge Massachusetts USA; ^10^ Department of Clinical Pharmacy Faculty of Pharmacy, Assiut University Assiut Egypt; ^11^ Amsterdam Cardiovascular Sciences Research Institute Amsterdam UMC Amsterdam the Netherlands

**Keywords:** long COVID, multi‐omics, phenotypes, post‐acute sequelae of COVID‐19, post‐viral condition

## Abstract

An estimated 10% of coronavirus disease (COVID‐19) survivors suffer from persisting symptoms referred to as long COVID (LC), a condition for which approved treatment options are still lacking. This systematic review (PROSPERO: CRD42024499281) aimed to explore the pathophysiological mechanisms underlying LC and potential treatable traits across symptom‐based phenotypes. We included studies with primary data, written in English, focusing on omics analyses of human samples from LC patients with persistent symptoms of at least 3 months. Our search in PubMed and Embase, conducted on January 8, 2024, identified 642 studies, of which 29 met the inclusion criteria after full‐text assessment. The risk of bias was evaluated using the Joanna Briggs Institute appraisal tool. The synthesis of omics data, including genomics, transcriptomics, proteomics, metabolomics, and metagenomics, revealed common findings associated with fatigue, cardiovascular, pulmonary, neurological, and gastrointestinal phenotypes. Key findings included mitochondrial dysfunction, dysregulated microRNAs associated with pulmonary dysfunction, tissue impairment, blood–brain barrier disruption, coagulopathy, vascular dysfunction, microbiome disturbances, microbial‐derived metabolite production and persistent inflammation. Limitations include cross‐study heterogeneity and variability in sampling methods. Our review emphasizes the complexity of LC and the need for further longitudinal omics‐integrated studies to advance the development of biomarkers and targeted treatments.

## 1 Introduction

Coronavirus disease (COVID‐19) caused the most recent pandemic with high incidence and mortality rates. While today the virus seems to cause less severe disease, the long‐term health effects persist [1]. Chronic symptoms post‐infection, known as Long COVID (LC), post‐acute sequelae of COVID‐19, or post‐COVID‐19 condition manifest in ~10% of COVID‐19 cases [2]. Most LC patients, despite negative polymerase chain reaction (PCR) tests, experience delayed recovery [3]. A clear definition of LC is lacking, although the World Health Organization (WHO) describes it as symptoms lasting for > 12 weeks post‐infection, but it is also described as the persistence of at least one symptom for more than 4 weeks after the clearance of SARS‐CoV‐2 without the presence of an alternative diagnosis [4, 5].

LC is multi‐systemic, affecting multiple organs, resulting in a broad array of symptoms that can be categorized into fatigue, respiratory, neurological, cardiovascular and gastrointestinal complaints [6, 7, 8]. Globally, observational studies aim to elucidate LC mechanisms and identify treatable traits [6, 7, 8], but clinical trials and efforts to repurpose drugs remain limited, while treatment options are desperately needed [9, 10].

LC's complexity and heterogeneity demonstrate the need for precision medicine, requiring an understanding of the interplay of biomolecules. The analysis of various biological molecules in large quantities simultaneously has revolutionized our ability to gain deep knowledge in biological systems on many scales. Combined data from various omics layers, including the (epi)genome, transcriptome, proteome, metabolome, and metagenome, could provide novel insights into the pathophysiology of LC, in association with the reported symptoms or phenotypes. This integrated knowledge holds the potential to drive target and drug discovery efforts that can ultimately be translated to personalized prevention and treatment strategies in LC. However, assessing large‐scale multi‐omics data is challenging and requires multidisciplinary approaches [11, 12, 13]. The objective of this study was to systematically review the literature to assess how omics‐based analyses across various symptom‐based phenotypes contribute to understanding pathophysiological mechanisms and identifying potential biomarkers and treatable traits relevant to precision medicine in LC patients compared with recovered individuals or healthy controls.

## 2 Methods

### 2.1 Protocol and Registration

Prior to the start of this systematic review, a detailed protocol was developed and registered with the International Prospective Register of Systematic Reviews. (PROSPERO, CRD42024499281). This review was conducted following a protocol in accordance with the PRISMA 2020 statement [14].

### 2.2 Search Strategy

Initial, limited searches were performed in PubMed to identify relevant key words and index terms, which resulted in the final search term (Figure S1). The search syntax was then adapted for Embase (Figure S2). The final search was performed by N.B.

### 2.3 Inclusion and Exclusion Criteria

The inclusion criteria for papers were: (1) LC defined as the persistence of symptoms post‐COVID for at least 3 months, consistent with the WHO definition [15], (2) inclusion of primary data, (3) omics analyses had to be based on human samples, and (4) subjects had to be alive at the time of sample collection.

Exclusion criteria for papers were: (1) not written in English, (2) abstracts only, (3) preprints, and (4) retracted papers. Studies identified through the systematic search underwent title, abstract screening, and full‐text eligibility assessment, performed by N.B. and EMAS, after which relevant studies were included in this systematic review. There were no restrictions concerning study criteria for group sizes or study design.

### 2.4 Quality and Risk of Bias Assessment

Following title and abstract screening, a quality and risk of bias assessment was performed independently by two reviewers (N.B. and E.M.A.S.), using the 2020 revised critical appraisal instruments from the Joanna Briggs Institute [16]. Disagreements were discussed with a third reviewer (K.G.).

### 2.5 Data Extraction

Data extraction was conducted systematically by two independent reviewers (N.B. and E.M.A.S.) to ensure consistency and accuracy, with discrepancies resolved through discussion or consultation with a third reviewer (K.G.). For each study, we extracted data on the lead author, publication year, study design, patient characteristics, sample size per group, biological sample type (such as blood, urine etc.), timeframe for LC (in months post‐infection), omics layers, sample collection time points (post‐infection), outcome parameters (omics outcomes such as pathways and other parameters such as cytokine measurements or medical data) and omics analysis platforms. Findings were categorized by the omics layers, such as genomics, transcriptomics, proteomics, metabolomics, or metagenomics. Longitudinal data were extracted where applicable, and we considered whether studies controlled for confounding factors.

### 2.6 Data Analysis, Synthesis and Reporting

Data analysis, led by N.B. and reviewed with the team, systematically synthesized omics findings on LC. A qualitative synthesis identified recurring themes, patterns, and trends across omics layers, categorized by common LC symptom‐based phenotypes. While the classification of symptom‐based phenotypes may be somewhat arbitrary, we focused on key categories frequently mentioned in relation to LC symptoms, including fatigue, pulmonary, neurological, cardiovascular, and gastrointestinal symptoms [6, 7, 8, 17, 18]. Results were presented in tables, figures, and narrative summaries to provide a comprehensive overview of omics signatures in LC, outlining key findings, implications, and recommendations for future research.

## 3 Results

**FIGURE 1:**
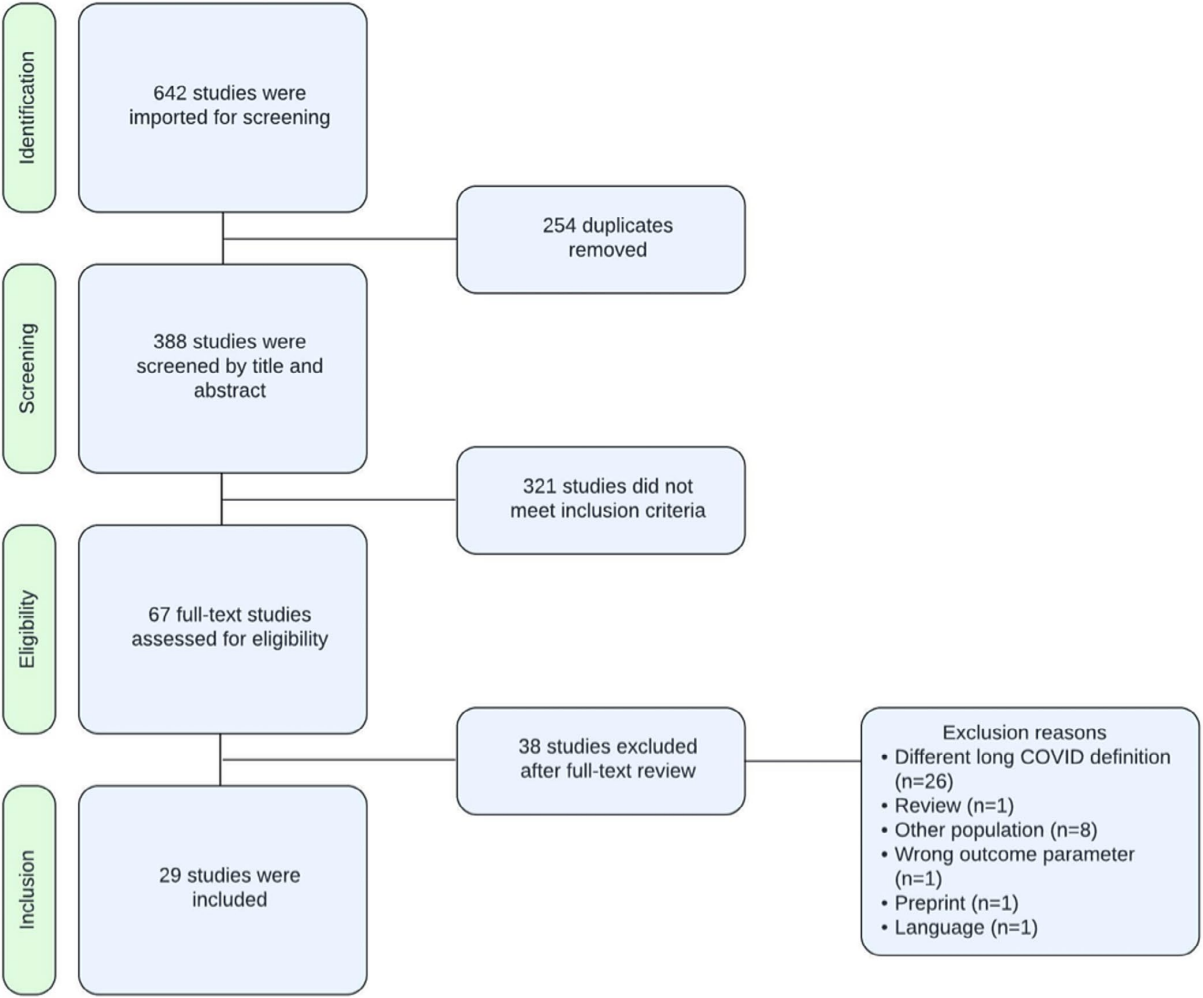
Flow diagram of included studies in this systematic review according to the PRISMA 2020 statement [14].

**FIGURE 2:**
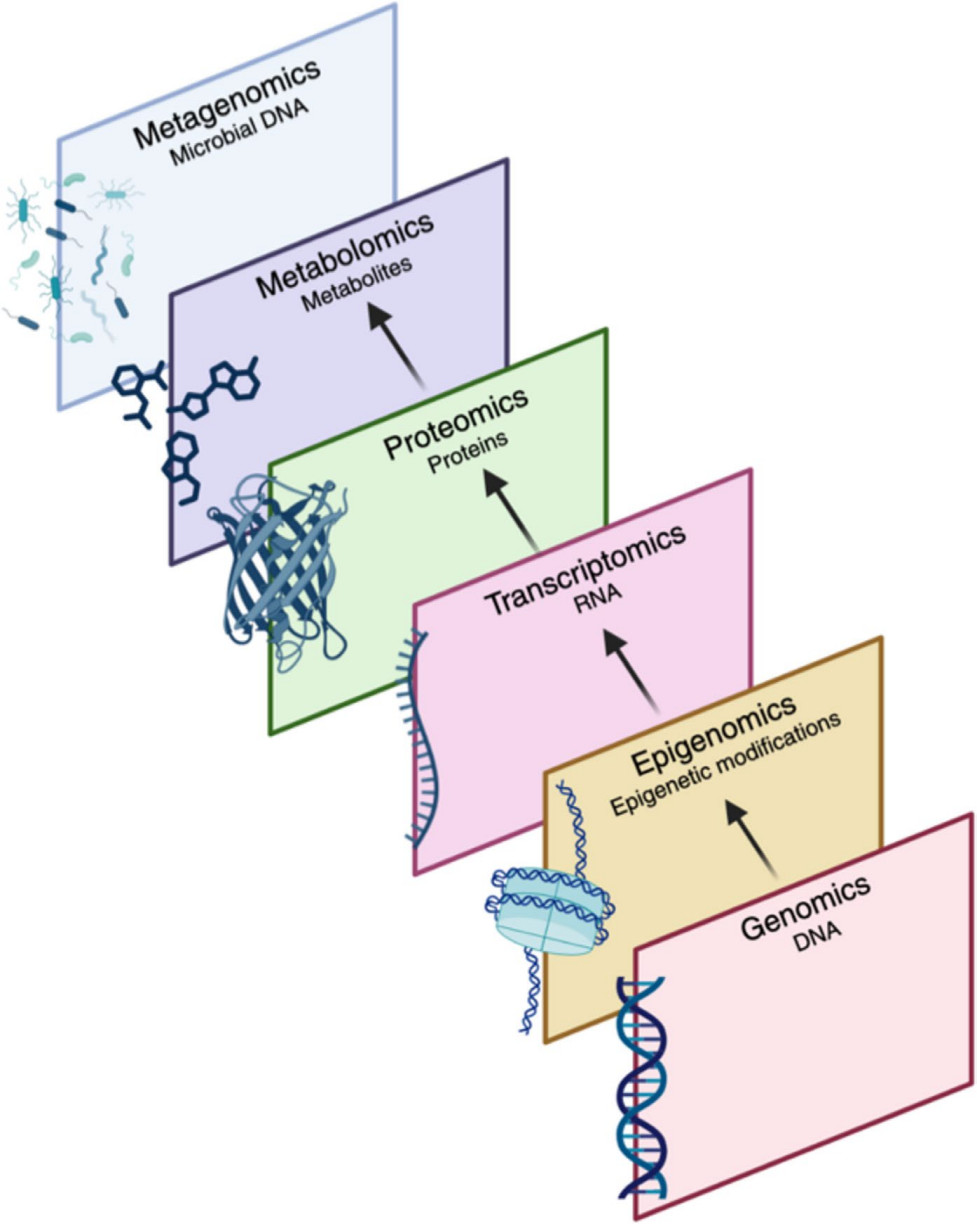
Omics layers included in this systematic review and the totality of biomolecules that they measure in a biological sample. The arrows illustrate how genomics to metabolomics are connected; DNA undergoes epigenetic modifications regulating gene activity, RNA transcription produces RNA transcripts, which are consequently translated into proteins that perform various cellular functions, ultimately driving metabolic processes and resulting in metabolite production.

On 8 January 2024, the search yielded 642 results, all of which were screened for eligibility according to the inclusion and exclusion criteria. Figure 1 provides an overview of the number of studies that were found to be suitable for further exploration and analysis, following a critical appraisal (Tables S1–S3). Quality assessment across cross‐sectional, cohort, and case–control studies indicated generally high methodological rigor, with all studies meeting key criteria such as reliable outcome measurement and appropriate statistical analysis. Microbiome studies utilized the shotgun metagenomics technique, which includes the totality of the microbial genomes to study the microbiome. Figure 2 provides additional clarification on the studied omics layers. Table 1 shows a description of the general characteristics of included studies, and Table S4 includes additional extracted data on LC sample collection time points, outcome parameters, and omics analysis platforms.

**TABLE 1:** General characteristics of the included studies.

Study (lead author, year of publication)	Type of study	Long COVID patient characteristics*	Sample size	Biological sample type for omics analysis	LC timeframe	Omics layer(s)
2021						
Zhao et al. 2021 [19]	Cross‐sectional	Age, years (mean ± SD): 52 ± 13 Female, *N* (%): 17 (77%) AC disease severity: mild 90.1%, moderate 9.1%, severe 4.5%	22 LC 22 PC 22 HC	Plasma	3 months	Proteomics
2022						
Liu et al. 2022 [20]	Cohort	Age, years (median, IQR): 48.7 (33–62) Female, *N* (%): 46 (56.8%) AC disease severity: Asymptomatic 4.9%, Mild 28.4%, Moderate 51.9%, Severe 8.6%, Critical 6.2%	81 LC 106 AC 68 HC	Feces	6 months	Metagenomics
Kruger et al. 2022 [21]	Cross‐sectional	Age, years (median, range): High responder: 51 (40–60) Low responder: 45 (31–58) Female, *N* (%): 69 (70%) AC disease severity: mild 71%, moderate 5%, severe 17%, 7% unknown	99 LC (2 groups) 23 HC	Plasma (platelet‐poor)	7 months	Proteomics
Ryan et al. 2022 [22]	Cohort	Convalescents including LC Age, years (median, range) 58 (23–77) Female, *N* (%): 34 (49%) AC disease severity: mild 72%, moderate 10%, severe 10%, critical 8%	21 LC 48 PC 14 HC	Blood	6 months	Transcriptomics
Vijayakumar et al. 2022 [23]	Cohort	Age, years (median, range): 56.5 (51.3–66.0) Female, *N* (%): 8 (21.1%) AC disease severity: mild 97.8%	38 LC 29 HC	BAL and plasma	3–6 months	Proteomics
2023						
Aschman et al. 2023 [24]	Case–control	Age, years, (mean ± SD): 45.1 ± 11.5 Female, *N* (%): 1 (9%) AC disease severity: mild 91%, severe 9%	11 LC 8 C 8 HC	Vastus lateralis muscle biopsy and serum samples	6 months	Transcriptomics, proteomics
Berezhnoy, et al. 2023 [25]	Cross‐sectional	Age, years, (mean ± SD): 56.9 ± 14.9 Female, *N* (%): 16 (48.5%)	7 AC 33 LC 12 PC 73 HC	Plasma, serum	3 months	Metabolomics, proteomics
Dufrusine, et al. 2023 [26]	Cross‐sectional	Age years (mean ± SD): 62.7 ± 13.2 Female, *N* (%): 5 (50%) AC disease severity: mild–severe	10 LC 30 AC 25 HC	PBMCs	3–6 months	Proteomics
Guo et al. 2023 [27]	Case–control	Age years (median, IQR): 43.00 (28.00, 50.00) Female, *N* (%): 7 (77,8%)	9 LC 14 PC	PBMCs	3 months	Metabolomics
Iosef, et al. 2023 [28]	Cross‐sectional	Age, median (IQR): 61.0 (20.5) Female, *N* (%): 10 (45,5%)	22 LC 44 AC 22 HC	Plasma	3 months	Proteomics
Kazantseva et al., 2023 [29]	Case–control	Age, years (range): 18–25 Female, *N* (%): 64 (79.0%) AC disease severity:mild–moderate disease course	81 LC 225 AC 61 PC 111 HC	Blood (leuko‐cytes)	6–12 months	Genomics
Kovarik et al., 2023 [30]	Cross‐sectional	Age, years (mean, range): 33 (21–53) Female, *N* (%): 9 (69.2%)	13 LC 13 PC 12 HC	Plasma	3 months	Proteomics, metabolomics
López‐Hernández et al. 2023 [31]	Cohort	Age, years (median, Q1‐Q3): 51.5 (43.5–60.8) Female, *N* (%): 20 (41.7%) AC disease severity: Mild 12.5%, moderate/severe 77%, critical disease 10.4%	15 AC 48 LC 37 HC	Plasma	24 months	Metabolomics
Mahdi et al. 2023 [32]	Cross‐sectional	Age, years (mean ± SD): LC (POTS): 39 ± 11 LC: 44 ± 11 Female, *N* (%): LC (POTS): 19 (95%) LC: 22 (100%)	20 LC (POTS) 22 LC 21 HC	Plasma	6 months	Proteomics
Medori et al. 2023 [33]	Case–control	Age, years: > 18 Female: NA	80 LC 50 HC	Serum	6 months	Proteomics
Peppercorn et al. 2023 [34]	Cross‐sectional	Age, years (median): 39 Female, *N* (%): 5 (83.3%)	6 LC 9 ME/CFS 5 HC	PBMCs	12 months	Proteomics
Sanhueza et al. 2023 [35]	Cohort	Age (years), (SD): CT: 48.9 ± 10.3 DLCOc: 44.8 ± 10.5 CT ± DLCOc: 56.8 ± 11.9 Female, *N* (%): CT: 9 (39.1%) DLCOc: 4 (80%) CT ± DLCOc: 8 (57.1%) AC disease severity: CT: mild 13%, moderate 21.7%, severe/critical 65.2% DLCOc: mild 60%, moderate 40% CT ± DLCOc: mild 7.1%, moderate 42.8%, severe/critical 50%	42 LC (3 groups) 18 PC	Serum	4 months	Proteomics
Sykes et al. 2023 [36]	Cohort	Age years (mean, SD): 57.1 ± 10.2 Female, *N* (%): 13 (48%) AC disease severity (WHO score): Hospitalized, no oxygen therapy 26% Oxygen by mask or nasal prongs 44% Non‐invasive ventilation 7% Mechanical ventilation 22%	27 LC 10 HC	Small resistance arteries isolated from gluteal subcutaneous biopsy	3 months	Transcriptomics
Taenzer et al. 2023 [37]	Cross‐sectional	Age, years (median): 38 Female, *N* (%): 20, (80%)	25 LC 8 ME/CFS 8 HC	Urine	6 months	Metabolomics
Taylor et al. 2023 [38]	Case–control	Age, years (median, IQR): Severe: 45 (37–54) Fatigue: 45 (37–54) Female, *N* (%): Severe: 329 (71.7%) Fatigue: 355 (74.4%)	Severe 459 LC 864 PC Fatigue 477 LC 909 PC	Saliva	3 months	Genomics
Visvabharathy et al. 2023 [39]	Cohort	Age, years (mean, range): 46.6 (18–83) Female, *N* (%): 62 (66.0%) AC disease severity: mild 82.0%	94 LC 44 PC 34 HC	Plasma	6 months	Proteomics
Wang et al. 2023 [40]	Cohort	Age, years (median, IQR): Mild: 62 (44–73) Severe: 62 (57–73) Female (*N*, %): Mild: 12, (37.5%) Severe: 28, (50.9%)	87 LC (2 groups) 30 PC 28 HC	Plasma	6 months	Proteomics, Metabolomics
Zhang et al. 2023 [41]	Cohort	Age, years (mean, range): 40.5 (23–68) Female, *N* (%): 18 (40.0%)	45 LC 8 PC 25 HC	Oral cavity specimen, feces, serum	3 months	Metagenomics, metabolomics
2024						
Brīvība et al. 2024 [42]	Cohort	Age, years (mean, ± SD): 53.31 ± 15.88 Female, *N* (%): 80 (41.1%)	146 AC (78 LC) 110 HC	Feces	3 months	Metagenomics
Cervia‐Hasler et al. 2024 [43]	Cohort	Age years (median, IQR): 36 (28–59) Female, *N* (%): 33 (45.2%) AC disease severity: severe 16.4%	113 AC 40 LC 39 HC	Serum, PBMCs	6 months	Proteomics, transcriptomics
García‐Hidalgo et al. 2024 [44]	Cohort	Age, year (median, IQR): 63.0 (57.0; 68.0) Female, *N* (%): 7 (22.6%)	119 AC 31 LC 26 PC	Plasma	3 months	Transcriptomics, proteomics
Greene et al. 2024 [45]	Cohort	Age, years (mean): LC brain fog(−): 38 LC brain fog(+): 46 Female, *N* (%): LC brain fog(−): 9 (81.8%)LC brain fog(+): 11 (100%) AC disease severity: mild 56.6% Moderate: 13.1% Severe: 30.3%	76 AC 22 LC (2 groups) 10 PC 25 HC	PBMCs	3 months	Transcriptomics
Hamrefors et al. 2024 [46]	Cross‐sectional	Age, years (range): 18–70 Female, *N* (%): 26 (76.5%)	32 LC 27 POTS 39 HC	Feces	3 months	Metagenomics
Saito et al. 2024 [47]	Cross‐sectional	Age, years, (mean ± SD): 51.9 ± 12.2 Female, *N* (%): 6 (18.6%)	15 AC 30 LC 15 PC 15 HC	Plasma	12 months	Metabolomics

Abbreviations: AC, acute COVID‐19; BAL, bronchoalveolar lavage; C, type‐2b atrophy control cohort; CT, computed tomography; DLCO, diffusing capacity of the lungs for carbon monoxide; HC, healthy controls; LC, Long COVID; ME/CFS, myalgic encephalitis/chronic fatigue syndrome; PBMCs, peripheral blood mononuclear cells; PC, post‐COVID; POTS, postural orthostatic tachycardia syndrome.

**FIGURE 3:**
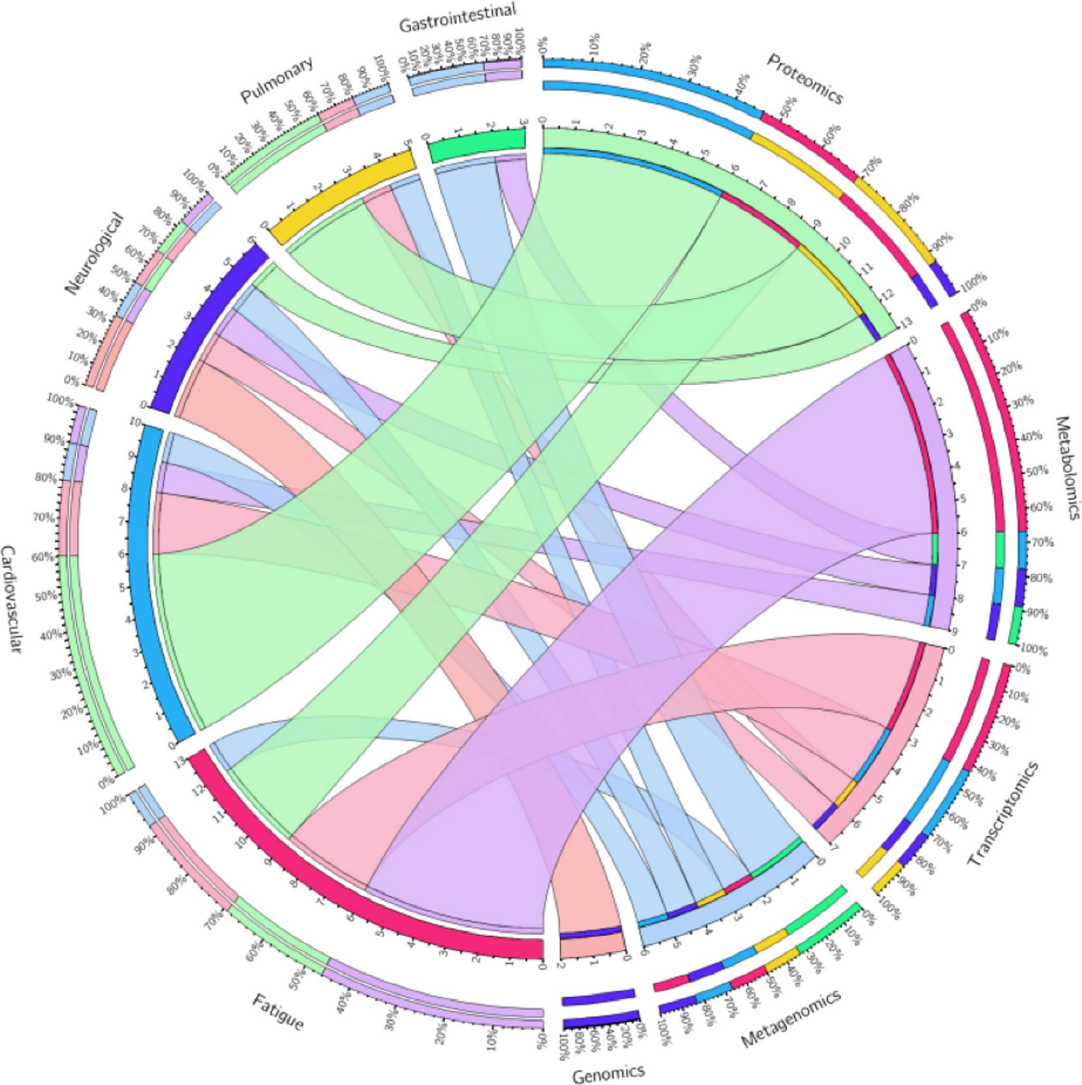
The frequency of omics findings related to LC symptom‐based phenotypes. The symptom‐based phenotypes and omics layers are each represented with a variety of bright and light colored bands, respectively. Percentages in the outer circle show to what extent studies involving specific omicslayers or phenotypes were included in this systematic review. This figure was created with Circos Table Viewer v0.63–10 from the Genomes Science Centre.

While omics results in this review are categorized according to symptom‐based phenotypes, it is important to acknowledge that symptoms in LC typically do not occur in isolation and may affect multiple organ systems simultaneously. This suggests that the underlying mechanisms contributing to LC are complex and interconnected, often manifesting in multiple phenotypes across the body. Figure 3 illustrates what efforts of omics studies related to the symptom‐based phenotypes were made and shows that, until now, most studies have focused on proteomics and metabolomics for cardiovascular and fatigue symptoms, respectively.

### 3.1 Fatigue and Altered Energy Metabolism

Post‐COVID fatigue is among the most commonly reported symptoms in LC [17].

#### 3.1.1 Metabolomics and Proteomics

Saito et al. identified distinct metabolomics profiles in LC patients with myalgic encephalitic/chronic fatigue syndrome (ME/CFS) symptoms a year post‐COVID, compared with controls, showing altered amino acid and energy metabolism pathways [47]. López‐Hernández's study revealed 53 and 27 metabolites to be dysregulated compared to acute COVID‐19 (AC) patients and controls, respectively [31]. LC patients could be distinguished according to lactic acid, lactate/pyruvate, ornithine/citrulline, and arginine [31]. Changes indicated mitochondrial dysfunction and redox imbalances in LC patients 2 years post‐infection [31]. Nuclear magnetic resonance (NMR) metabolomics indicated variations in lactate, pyruvate, glucose, and other metabolites between LC and AC patients, suggesting changes in energy metabolism [25]. Furthermore, LC patients had higher levels of citrate, histidine, and ornithine compared to AC and healthy controls (HC) [25]. The study explored the role of gender and age on their findings and found a strong gender‐age effect on acetate levels in LC [25].

In peripheral blood mononuclear cells (PBMCs), IDO2 or indoleamine 2,3‐dioxygenase‐2 was upregulated in LC patients and associated with mitochondrial dysfunction, resulting from reduced basal and maximal oxygen consumption rates, and with reduced intracellular Krebs cycle‐related compound levels and amino acids. The aryl hydrocarbon receptor was correlated with the induction of IDO2 and was therefore proposed as a possible therapeutic target [27]. Iosef et al. also demonstrated the increased levels of IDO2 in plasma proteomic samples [28]. The expression of IDO2 is moreover linked to decreased levels of tryptophan and increased kynurenine quantities, which have been proposed to cause fatigue symptoms [48]. Taenzer et al. reported a lower kynurenine/tryptophan ratio in LC patients with fatigue, with decreased levels of kynurenine and tryptophan [37]. Furthermore, this study also reported reduced levels of other amino acids, including phenylalanine and tyrosine [37]. The authors hypothesized the lower kynurenine/tryptophan ratio to be caused by impaired gut function or IDO function [37].

Targeted plasma metabolomics found low levels of amino acids and triglycerides, deregulated acylcarnitines, and defects in lipid oxidation, as well as elevated anti‐inflammatory osmolytes like taurine and hypaphorine [30]. The same study found protein alterations related to macrophage‐derived secreted proteins and fewer acute phase proteins [30].

#### 3.1.2 Transcriptomics and Proteomics

Gene expression changes in muscle biopsies of LC patients indicated matrix remodeling, angiogenesis, and immune dysregulation linked to interferons (IFN), type I and III, and the complement system, alongside decreased mitochondrial activity and energy production capacity. The study reported no sex‐related influence in their principal component analysis. Moreover, increased serum protein levels related to complement pathways were found in LC patients compared to controls [24].

LC and ME/CFS exhibited similar protein profiles, including B2M, BCL2, CD4, and HLA‐DRB1 [34]. Clustering of 162 differentially regulated proteins in LC revealed main clusters related to viral infections (SARS‐CoV‐2, Epstein–Barr) and immune responses (natural killer cell cytotoxicity, Fc epsilon R1 signaling) [34]. Mitochondrial dysfunction pathways were also affected, leading to reduced energy production and potential oxidative stress [34].

#### 3.1.3 Genomics and Transcriptomics

Genomics linked LC fatigue risk factors to metabolic/JNK signaling and genes shared with ME/CFS, involving insulin and circadian rhythm regulation. Single nucleotide polymorphisms (SNPs) more abundant in LC than ME/CFS were related to *ATP9A*, *CLOCK*, *SLC15A14*, *INSR*, and *GPC5*. *CLOCK* regulates circadian rhythm and mitochondrial function [38]. Thrombocytopenia has been linked to post‐viral fatigue complaints. A longitudinal transcriptomics analysis in the blood of COVID‐19 convalescents by Ryan et al. revealed distinct profiles in individuals that developed LC. In the transcriptome of LC patients, downregulation of genes related to platelet factor 4, platelet glycoprotein IX, thrombopoietin receptor, and coagulation in platelet‐related pathways was found at 6 months post‐COVID [22].

#### 3.1.4 Metagenomics

From a microbiome perspective, in fecal samples that were collected 6 months post‐COVID, *Clostridium innocuum* and *Actinomyces naeslundii* were significantly associated with fatigue symptoms [20].

Together, these studies indicate associations between LC fatigue symptoms and neuroinflammation, mitochondrial dysfunction, alterations in lipid metabolism, thrombocytopenia, and microbiome disturbances.

### 3.2 Pulmonary

Commonly reported respiratory symptoms in LC include shortness of breath and cough [17].

#### 3.2.1 Transcriptomics and Proteomics

Plasma profiling identified microRNAs, miR‐9‐5p and miR‐486‐5p, linked to impaired lung diffusion capacity (< 80%) and involved in inflammation, angiogenesis, tissue repair, and cell senescence pathways. RNA sequencing and proteomics further emphasized these pathways in patients with pulmonary dysfunction, who were mostly older patients with a relatively more severe disease course. As part of the targetome of both miRNAs, key upregulated genes included *PXDN*, *ITGA2*, *MXRA7*, *TMEM200A*, *RNF187*, and *FAT1*, while downregulated genes included *NELL2*, *RAI14*, *SLC24A2*, *PPP1R9A*, and *CHRM3*. Identified proteins, PTN and KIM1, are key players in tissue repair pathways [44].

Proteomics studies on blood and airway samples from LC patients with persistent radiological abnormalities 3–6 months post‐discharge revealed abnormal airway proteomes by the presence of CASP3, EPCAM, KRT19, and TGFA, indicative of epithelial injury and apoptosis. These disruptions in tissue repair pathways were absent in HC [23]. Additionally, increased numbers of cytotoxic lymphocytes were associated with epithelial damage and impaired lung function, emphasizing the immune system's role in pulmonary phenotypes [23]. From an immunological perspective, proteomics studies found that pulmonary dysfunction in LC at 4 months post‐infection was associated with upregulated pathways of chemotaxis of phagocytes, leukocyte activation, and cardiac dysfunction, and with elevated levels of chemokine CXCL9 during AC [35]. Furthermore, correlations were found between serum levels of glucocorticoid receptor and dyspnea in LC patients [33].

#### 3.2.2 Metagenomics

Persistent pulmonary symptoms were correlated with increased abundance of opportunistic gut pathogens such as *streptococcus anginosus* , *Streptococcus vestibularis* , *Streptococcus gordonii* , and *Clostridium disporicum* in fecal samples collected at 6 months post‐COVID [20].

These findings collectively demonstrate that in pulmonary LC, miRNA dysregulation was associated with impaired diffusion capacity. Findings also indicated potential impairments in tissue repair mechanisms and ongoing immune responses, including dysregulated CD8^+^ T cell responses associated with pulmonary abnormalities.

### 3.3 Neurological

Neurological manifestations can include cognitive impairment (brain fog), memory deficits, and also neuropsychiatric symptoms, defining the involvement of the central nervous system in LC [7].

#### 3.3.1 Proteomics and Genomics

LC patients with neurologic complaints exhibit a distinctive plasma proteomic profile compared to COVID‐19 convalescents, characterized by reduced pro‐inflammatory and antiviral response proteins, alongside increased immunoregulatory proteins, correlating with neurocognitive dysfunction [39]. Genetic associations were identified between SNPs and neurological symptoms, in which *FURIN* and *SLC6A20* were emphasized [29]. Taylor et al. identified 73 genes related to severe LC and fatigue‐dominant cohorts, with five genes (*D2HDGH*, *GUCY1A2*, *PCSK2*, *CCDC146*, *PGPEP1*) involved in metabolism and regulatory pathways present in both. Combinatorial analytics highlighted 42 genes as potential therapeutic targets, with 13 already linked to existing drugs. Pathway analysis showed these genes were mostly related to neurological and cardiometabolic diseases. TLR4 was proposed for drug repurposing [38].

#### 3.3.2 Transcriptomics

Brain fog, a neurological manifestation of LC, can lead to cognitive impairment. Greene et al. found significantly increased concentrations of S100β, a marker of blood–brain barrier (BBB) dysfunction that was also positively associated with age, in AC patients. A transcriptomics study in LC patients with brain fog revealed differentially expressed genes compared to recovered individuals that showed dysregulated immune responses. Upregulated genes were related to T‐cell activation, angiogenesis (including *HIF1A*, *HES1* and *DLL1*) and TGF‐signaling (including *SMAD3*, *SNAI1*, *SMURF1*), crucial for BBB regulation. Downregulated genes involved platelet activation and hemostasis, suggesting systemic alterations and vascular abnormalities. Compared to LC patients without brain fog, those affected had distinct gene expression profiles related to T‐cell differentiation and activation (including *TNFSF9*, *IFNG* and *RUNX3*), but also the negative regulation of immune responses, pointing to sustained systemic inflammation (including *WASL*, *TNFAIP3* and *ID2*) and BBB disruption [45].

#### 3.3.3 Metagenomics and Metabolomics

A higher abundance of *Clostridium innocuum* and *Actinomyces naeslundii* in LC patient's fecal samples is linked to neuropsychiatric symptoms, suggesting a microbial component to LC's neurological impact [20]. Saito et al. reported an inverse correlation between reduced plasma metabolite sarcosine and LC symptoms, cognitive dysfunction, and depression scores. Similarly, lower serine levels were inversely associated with anxiety and depression in LC patients with ME/CFS [47]. Taenzer et al. reported altered concentrations of serotonin, catecholamines, and dopamine in LC [37].

Collectively, these findings illustrate that persistent systemic inflammation, BBB disruption, vascular abnormalities, microbiome imbalances, and metabolic changes are related to the spectrum of neurological symptoms experienced during LC.

### 3.4 Gastrointestinal

Brīvība et al. stated that while COVID‐19 is a respiratory condition, 20%–50% of LC patients experience gastrointestinal symptoms [42]. Nausea, diarrhea, and loss of appetite are common gastrointestinal symptoms linked to LC [17].

#### 3.4.1 Metagenomics

Research indicates a decreased alpha and beta diversity of the gut microbiome, as evidenced by data from fecal samples collected 3 months post‐COVID among patients with persistent gastrointestinal symptoms [41]. This decreased relative abundance at the phylum level included *Proteobacteria*, *Fusobacteria*, *Spirochaetes*, *Actinobacteria*, and *Uroviricota*. Moreover, based on stool and saliva samples of these LC patients, ectopic colonization of the oral cavity by gut microbes was detected by 27 differentially abundant genera of the *Proteobacteria* phylum. Among these, bacteria such as *Neisseria*, *Lautropia*, and *Agrobacterium* were linked to potentially harmful serum metabolites, including estradiol valerate, 5‐sulfoxymethylfurfural, and 4‐chlorophenylacetic acid [41]. At 3 months post‐infection, changes in gut microbiome composition included an increased abundance of butyrate‐producing bacteria like *Roseburia* and *Faecalibacterium prausnitzii* . This study also emphasized that antibiotic use during AC infection could alter bacterial abundance. Moreover, lower alpha diversities during the acute infection were related to LC compared to recovered individuals. Notably, the recovered group had an increased abundance of *Prevotella* species, potentially revealing a protective effect. However, among the included patients in this study, it remained unclear how many participants experienced gastrointestinal symptoms [42].

So far, microbiome imbalances have been identified, and toxic metabolite production has been associated with gastrointestinal health.

### 3.5 Cardiovascular

Cardiovascular symptoms such as palpitations and chest pain in LC patients suggest ongoing pathologies [49].

#### 3.5.1 Transcriptomics and Proteomics

At 3 months post‐COVID, gene expression analysis revealed that upregulation in pathways related to proteoglycan synthesis, extracellular matrix alteration, and viral mRNA replication was related to vascular smooth muscle cell dysfunction and vascular fibrosis development in LC [36]. Rho‐kinase inhibitors, targeting these changes, are proposed as potential therapeutic options for restoring vascular homeostasis [36].

A plasma proteomics study compared LC patients to HC and AC subjects. LC patients showed a shift in natural killer cell phenotype from active to resting and neutrophils forming extracellular traps, linked to vascular alterations mediated by angiopoietin‐1 and VEGF‐A. Activation of TGF‐β, TNF‐α, and HIF‐1 pathways indicated systemic inflammatory responses. This profiling suggests cardiovascular and potential neurologic dysfunction due to protein expression patterns affecting neuronal and cognitive functions [28].

Other proteomic findings suggest changes in iron metabolism proteins including transferrin, hemopexin, lipocalin, superoxide dismutase 1, and ceruloplasmin in LC. Furthermore, it was found that lipoxygenase was activated, possibly through an iron‐dependent post‐translational mechanism. The authors hypothesize that leukotriene (LT) B4 and lipocalin 2 could be explored as novel targets for the treatment and prevention of LC [26].

Longitudinally, Cervia‐Hasler et al. found evidence of increased complement activation during the acute infection phase, which persisted in LC patients. Additionally, proteome analysis identified thrombo‐inflammatory markers in blood samples at 6 months, including elevated levels of von Willebrand factor (VWF), suggesting endothelial activation and red blood cell lysis. In patients where LC symptoms persisted for 12 months, increased monocyte‐platelet aggregates and platelet activation markers were observed at 6 months, indicating a pro‐thrombotic environment [43].

#### 3.5.2 Proteomics and Metabolomics

A proteomics study analyzed the content of fibrin amyloid microclots and found dysregulated pro‐inflammatory markers involved in coagulation, cellular function, and lipid metabolism [21]. Specifically, elevated levels of von Willebrand factor (VWF) and increased platelet factor 4 activation were observed, while plasma kallikrein levels were reduced in long COVID patients compared to HC [21]. Additionally, the authors reported the presence of antibodies within the microclots, suggesting these could elicit an autoimmune response to persistent fibrin amyloid microclots [21]. Wang et al. identified increased plasma levels of microbiota‐derived metabolites such as trimethylamine and phenylacetylglutamine that were associated with cardiovascular disease in LC [40].

In LC patients with or without postural orthostatic tachycardia syndrome (POTS), which can also be regarded as a neurological condition, proteomic analysis showed similar protein dysregulation affecting coagulation/hemostasis, immune response, angiogenesis, and metabolism [32]. Key proteins included SERPINE, linked to increased clotting susceptibility, and CCL5, associated with hyper‐inflammation and T‐cell involvement, suggesting a shared pathophysiological profile [32].

#### 3.5.3 Metagenomics

Moreover, microbiome analyses revealed significant gut flora differences between POTS and LC patients compared to controls, with variations in microbial diversity and specific taxa potentially related to gastrointestinal and fatigue symptoms. LC was associated with alterations in the abundance of phyla *Ascomycota* and *Firmicutes* compared with controls [46]. Together, these findings show how cardiovascular symptoms in LC are related to vascular dysfunction, systemic inflammation and coagulation, and microbiome disturbances.

### 3.6 Persistent Involvement of the Immune System

While the presented results show that the different symptom‐based LC phenotypes are related to specific findings in the studied omics layers, the observed biomolecular signatures suggest significant overlap, such as mitochondrial and vascular dysfunction, and indicate that a dysregulated immune system is a common underlying factor.

Persistent systemic inflammation, characterized by elevated cytokine levels and T cell activation, affects multiple organ systems in LC. Elevated IL‐17 levels in LC patients' plasma 2 years post‐infection indicate ongoing immune activation [31]. IL‐17, primarily produced by T helper cells, is crucial during AC and is linked to chronic inflammatory diseases such as rheumatoid arthritis, psoriasis, and metabolic syndrome [50, 51]. These conditions share common pathologic IL‐17‐related characteristics such as vascular dysfunction and hypertension, increasing the risk of cardiovascular disease [52]. This suggests that persistently elevated IL‐17 levels in LC could similarly contribute to cardiovascular complications. In LC, elevated IL‐17 levels have moreover been correlated with pre‐existing hypertension, suggesting a complex interaction between chronic post‐COVID manifestations, including pulmonary fibrosis, and pre‐existing health conditions [53, 54]. Proteomic analysis further supported the role of immune dysregulation, showing significant upregulation of CXCL10 in LC patients relative to COVID‐19 convalescents and HC [19]. This finding is corroborated by Green et al. who also report elevated CXCL10 levels in blood samples, demonstrating a sustained IFN‐induced inflammatory response [45].

A proteomic study identified inflammation‐related proteins that could serve as potential biomarkers for LC, including Siruin 1, Natriuretic Peptide B, Hemopexin, and Arachidonate 5‐Lipoxygenase. Multivariate analysis demonstrated significant protein changes from HC. It was notable that findings also varied among LC patients, demonstrating the heterogeneity of the disease [33].

A persistent pro‐inflammatory state was identified in all symptom‐based phenotypes, indicating the importance of the role of the immune system in the development of LC.

### 3.7 Multi‐Omics and Long COVID

Several of the included studies have employed multi‐omics approaches, referring to the measurement of two or more omics layers from the same samples, to understand the interplay of biomolecules in LC. Serum proteomics identified increased activation of the complement system in the serum of LC patients by increased soluble C5bC6 complexes and decreased levels of C7‐containing terminal complement complexes. Other than innate immune responses, thrombo‐inflammation was seen. In single‐cell transcriptomic signatures of monocytes, *NR4A1* was decreased while interferon‐induced transcripts, including *IFITM3*, were increased, suggesting an inflammatory response [43]. Proteomics and metabolomics findings in a study by Wang et al. showed significant alteration in acute phase proteins and platelet degranulation that are indicators of inflammatory and immune responses, but also persistent dysregulation of metabolites of the TCA cycle and glucose metabolism that reflect adjustments to inflammation. Furthermore, both layers reflected metabolic dysregulation [40]. Other studies demonstrated overlapping findings on proteomics and metabolomics concerning energy imbalance and inflammatory alterations [25, 30]. Unsupervised clustering identified three distinct disease phenotypes based on the omics profiles, demonstrating the heterogeneity of LC. Cluster B was characterized primarily by a signature of triglycerides and organic acids, while cluster C, which included a higher proportion of women, was associated with molecules enriched in the HIF‐1⍺ pathway, known for gender‐specific differences [40]. García‐Hildago et al. found pathways of tissue repair including focal adhesion, MET signaling, and L1CAM interaction, and on a proteomic level, found increased proteins involved in tissue repair in patients with persistent pulmonary abnormalities [44]. Among the 73 genes that Taylor et al. identified in their genomics analysis, primarily related to neurological and cardiometabolic disease pathways, 14 were also differentially expressed in a transcriptomic study assessing whole blood from LC patients [38]. Zhang et al. correlated the presence of ectopic bacteria in the oral cavity with serum metabolites [41]. Together, studies utilizing multiple omics layers have demonstrated that examining various layers can confirm findings or provide additional context.

## 4 Discussion

**FIGURE 4:**
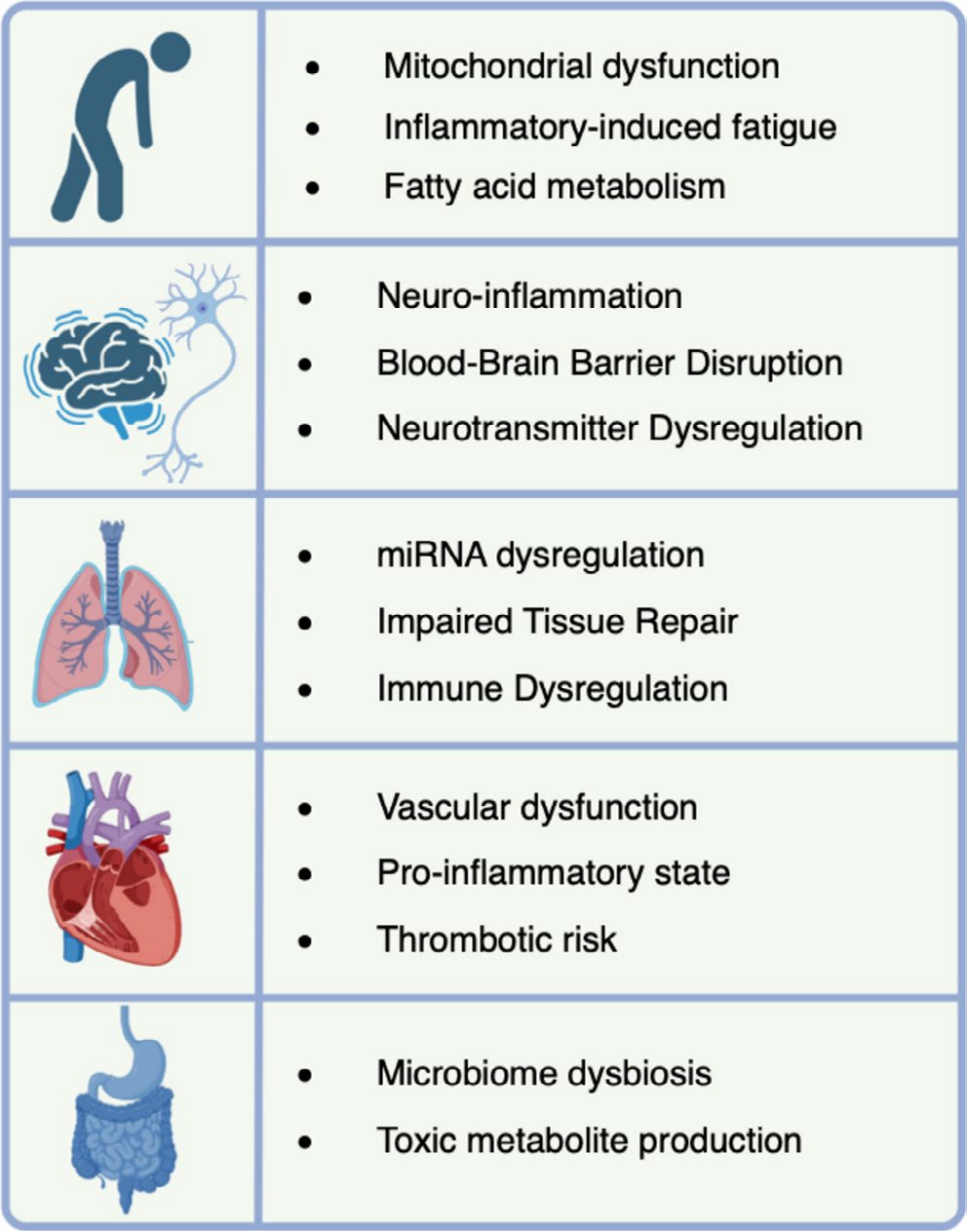
Treatable traits in long COVID stratified by phenotype. This figure summarizes the key biological disruptions identified in LC, such as mitochondrial dysfunction, neuroinflammation, and immune dysregulation, organized by symptom category (fatigue, neurological, pulmonary, cardiovascular, and gastrointestinal). Created with BioRender.com.

To our knowledge, this systematic review is the first to provide a comprehensive omics overview of symptom‐based phenotypes in LC, contributing to our understanding of its pathophysiology and identification of potential biomarkers and treatable traits. Our findings reveal that LC symptoms demonstrate a complex interplay of biomolecules, with inflammation and immune dysregulation as central mechanisms driving multi‐systemic effects across multiple organ systems. Figure 4 illustrates treatable traits for LC management; however, further validation is needed to determine the clinical significance of these findings.

Distinct biomolecular patterns are evident in LC symptoms across omics layers. Fatigue was related to neuro‐inflammation, mitochondrial dysfunction, and lipid metabolism imbalances. Mitochondrial dysfunction and inflammation, including the immunomodulatory IDO2, were observed in relation to altered energy metabolism and kynurenine metabolism in LC. Furthermore, altered fatty acid metabolism, including lipid oxidation, may affect overall energy homeostasis. Parallels are often drawn between LC fatigue and ME/CFS [55, 56, 57]; this condition can also be caused by other viral pathogens like influenza, enteroviruses, cytomegaloviruses, Epstein–Barr virus, and herpesviruses [58, 59, 60, 61]. In pulmonary LC, miRNA dysregulation was associated with impaired diffusion capacity, emphasizing the significance of long non‐coding RNA. Pulmonary symptoms were also associated with impaired tissue repair, which could eventually lead to fibrosis similar to that seen in idiopathic pulmonary fibrosis, potentially providing a guideline for its management [62]. Moreover, other aspects may play a role in the inflammatory phenotypes seen in pulmonary LC, such as neutrophil extracellular traps that were identified in patients with respiratory LC sequelae at 6 months post‐COVID [63]. Furthermore, mast cell activation was associated with immune dysfunction post‐COVID [64]. Gastrointestinal symptoms indicated microbiome imbalances and toxic metabolite production that can worsen gastrointestinal health. Targeting toxic metabolites, including estradiol valerate, 5‐sulfoxymethylfurfural, and 4‐chlorophenylacetic acid, could be targets to further investigate in this context. Overall, the scarcity of omics data related to gastrointestinal LC symptoms emphasizes the need for more research to better understand these associations and their implications for affected patients. In cardiovascular symptoms of LC, vascular dysfunction may play a role in changes in endothelial and smooth muscle function in which Rho‐kinase alterations are involved. A pro‐inflammatory state has also been related to cardiovascular phenotypes, including leukotriene B4, lipocalin 2, and the HIF‐1 pathway. Moreover, enhanced clotting tendencies were identified through immune and vascular alterations. Neurological symptoms showed blood–brain barrier disruption, neurotransmitter dysregulation, and persistent neuro‐inflammation through Notch signaling, TGF‐β, TNF‐and IFN pathways. Furthermore, upregulated CXCL10 levels were found in blood, indicating a sustained interferon‐induced response. While the involvement of the immune system is broad, cytokine levels may be altered, and human leukocyte antigen complex alterations (HLA‐DR) and IFN responses may also contribute to symptom persistence and severity. Compared to other viral respiratory infections, coronaviruses inhibit IFNs, causing a delayed type I and III response that can lead to uncontrolled viral replication and severe disease outcomes [65]. Severe COVID‐19 and LC are associated with elevated type I IFN‐α responses through cGAS‐STING pathway activation, contributing to sustained inflammation [66]. In LC, persistent inflammation has also been associated with autoimmunity, viral persistence, latent viral reactivation, and dysfunctional brainstem signaling [17].

This review has several limitations. Variability in study designs, timing of sample collection, and inconsistent LC definitions and timeframes complicate direct comparisons of data, currently precluding meta‐analysis. Limited omics data collection across various time points post‐infection limits the analyses of consistent LC stages. Additionally, factors like comorbidities such as diabetes or kidney disease, genetic predispositions, nutritional deficiencies, medication use, age, sex, reinfections, and exposure to different viral variants could have influenced findings, such as the levels of metabolites. Furthermore, the search strategy may not have captured all relevant studies.

The reported findings support the potential of precision medicine in LC management, using biomarkers and targeted treatments. To validate and expand on these findings, future studies should aim to provide longitudinal insights, include a broader range of LC subgroups, and ensure data accessibility for other researchers. Standardized protocols for sample collection and processing, as well as bioinformatics tools to minimize batch effects [67], would increase study robustness and facilitate stratified analyses by factors such as age, sex, comorbidities, and disease severity, and meta‐analysis. While the included studies provided valuable insights into LC at different time points, future studies focusing on samples collected at more standardized time points could offer a clearer perspective on the dynamics of omics layers during specific stages of the disease, improving consistency and advancing our understanding of the molecular changes occurring at different phases of LC. Furthermore, future studies may consider the longitudinal measurement of SARS‐CoV‐2‐specific IgG antibodies as a potential indicator of reinfection risk or underlying immune dysfunction. Declining SARS‐CoV‐2‐specific antibody levels were found within months after the initial infection [68]. Alterations, such as persistently elevated levels or delayed reduction, could indicate reinfection or persistent antigenic stimulation, both relevant to LC pathology [68]. Some of the current included studies measured SARS‐CoV‐2‐specific antibodies (Table S5), but not to assess reinfections. The measurements of PCR and antibodies before LC sample collection could increase the validity of findings in the context of reinfections [69]. Also, the impact of viral variants may be assessed to understand long‐term viral effects.

We focused on LC findings from (epi)genetic, transcriptomic, proteomic, metabolomics, and microbiome perspectives. Additional omics layers such as lipidomics, exposomics, and breathomics could provide further insights [32, 70]. Figure 2 outlines areas to improve validity and reveal biomolecule–phenotype associations. The majority of the included studies investigated proteomics and metabolomics, associated with cardiovascular fatigue symptoms, respectively, while epigenomics and metagenomics remain relatively unexplored. Several studies performed multi‐omics studies, showing how studying multiple layers can confirm and contextualize findings. Combining proteomics, metabolomics, and transcriptomics has shown promising results. Metagenomics and metabolomics revealed significant correlations that illustrated the effects of ectopic microbial colonization in the oral cavity. While different omics layers are related, their integration is still lacking in most studies. A published protocol by Wang et al. describes multi‐omics data analysis methods applicable to diseases other than LC, demonstrating efforts to help researchers understand how these data can be used to elucidate disease phenotypes in future studies [71].

While this was not the focus of this review, understanding the relationship between AC and LC from an omics perspective could be another valuable future direction to identify high‐risk individuals for LC development. AC reflects immediate changes in multiple omics layers, while LC is characterized by evolving and persistent alterations in these omics layers. Cai et al. reported that the burden of LC at 3 years post‐COVID is highest among previously hospitalized patients, demonstrating the impact of AC heterogeneity on LC [72]. Su et al. employed an integrated multi‐omics approach to identify LC biomarkers, emphasizing latent virus reactivation and autoantibodies as key factors, and classified patients on immune endotypes [73]. Stratification by acute disease severity has shown significant differences in the composition of the oral microbiome over time and the circulating proteome, linked to time after infection, as well as gene expression differences [22, 74, 75]. Furthermore, severe COVID‐19 was related to an increased risk of developing neuropsychiatric disorders, specifically Alzheimer's disease [76]. However, it is also suggested that the acute infection phase may not always provide insights into long‐term post‐viral effects [77].

## 5 Conclusion

This systematic review provided a comprehensive omics overview of LC symptom‐based phenotypes and offered recommendations for potential treatable traits. The findings demonstrate the progress in understanding the biomolecular mechanisms underlying LC and identify the next steps for future research. This will enhance our understanding of this complex condition and aid in developing targeted prevention and treatment strategies for precision medicine in LC.

## Author Contributions

All authors conceptualized the work, provided a critical review of the manuscript, and approved it for submission. N.B. searched for studies and drafted the review. N.B. and E.M.A.S. screened studies for eligibility and extracted data. N.B., E.M.A.S., K.G., and A.H.M.Z. were responsible for the methods.

## Conflicts of Interest

A.H.M.: She is the PI of a public‐private consortium (P4O2 (Precision Medicine for More Oxygen)) sponsored by Health Holland involving many private partners that contribute in cash and/or in kind (AbbVie. Boehringer Ingelheim, Breathomix, Clear, Fluidda, Ortec Logiqcare, Olive, Philips, Quantib‐U, Smartfish, Clear, SODAQ, Thirona, Roche, TopMD, Novartis, RespiQ). Received unrestricted research grant from GSK and Boehringer Ingelheim. Received Vertex Innovation Award Grant. Honoraria paid to the Institution from Boehringer Ingelheim, Astra Zeneca, and GSK. She is Chair of the DSMB of a study on BPD in neonates. K.G.: received funding from STIMAG, GSK ISS, ZonMw. Payments made to the institution by GSK and ALK. S.W.K.: received salary from the Cancer Research Institute, Postdoctoral Fellowship Grant. N.B., E.M.A.S., M.I.A.‐A., H.J.B.: nothing to declare.

## Supporting information


Data S1.

## Data Availability

Data sharing is not applicable to this article as no new data were created or analyzed in this study.
