# Supplementary material for: The Omics Landscape of Long COVID—A Comprehensive Systematic Review to Advance Biomarker, Target and Drug Discovery
Source: Allergy. 2025 Mar 14;80(4):932–48. doi: 10.1111/all.16526 (PMC11969314; doi:10.1111/all.16526)
Supplement: Supplementary file 1 — Data S1. [file ALL-80-932-s001.docx]

**Supplementary Material**

**Systematic search strategies**

Figure S1. PubMed search term.

(((((("Post-Acute COVID-19 Syndrome"[Mesh]) OR "after COVID*" OR "post-acute sequelae of SARS-CoV-2" OR "long-COVID*" OR "post-acute COVID* syndrome" OR "post acute COVID* syndrome" OR "months after SARS-CoV-2 infection" OR "post-acute sequelae of SARS-CoV-2 infection" OR "post acute sequelae of SARS-CoV-2 infection" OR "post-COVID*" OR "post COVID*" OR PASC OR "post-acute sequelae of COVID*" OR "post acute sequelae of COVID*" OR "post-COVID* condition*" OR "post COVID* condition*" OR "persistent* COVID*" OR "long-haul COVID*" OR "long haul COVID*" OR "Long-term COVID*" OR "chronic COVID* syndrome")) AND ((transcriptom*[tiab] OR metabolom*[tiab] OR proteom*[tiab] OR microbiom*[tiab] OR epigenom*[tiab] OR genom*[tiab]))) NOT ((Review[Publication Type]))) NOT ((pancrea*[tiab]))) NOT ((pairwise sequenc* comparison[tiab]))

Figure S2. Embase search term.

( ('after COVID*'/ OR 'post-acute sequelae of SARS-CoV-2'/ OR 'LC*'/ OR 'long-COVID*'/ OR 'post-acute COVID* syndrome'/ OR 'post-acute COVID* syndrome'/ OR 'months after SARS-CoV-2 infection'/ OR 'post-acute sequelae of SARS-CoV-2 infection'/ OR 'post-acute sequelae of SARS-CoV-2 infection'/ OR 'post-COVID*'/ OR 'post COVID*'/ OR PASC/ OR "post-acute sequelae of COVID*"/ OR "post-acute sequelae of COVID*"/ OR 'post-COVID* condition*'/ OR 'post COVID* condition*'/ OR 'persistent* COVID*'/ OR 'long-haul COVID*'/ OR 'long haul COVID*'/ OR 'Long-term COVID*'/ OR 'chronic COVID* syndrome'). ti,ab.) AND ( (transcriptom*/ OR metabolom*/ OR proteom*/ OR microbiom*/ OR epigenome*/ OR genom*).ti,ab.) NOT ((Review).ti,ab.) NOT ((pancrea*).ti,ab. ) NOT ((pairwise sequenc* comparison).ti,ab. )

**Critical Appraisal tools for use in JBI Systematic Reviews**

Table S1. Cross sectional studies

| Study | 1 | 2 | 3 | 4 | 5 | 6 | 7 | 8 |
| --- | --- | --- | --- | --- | --- | --- | --- | --- |
| Berezhnoy *et al*. 2023 | Yes | Yes | NA | Yes | NA | NA | Yes | Yes |
| Dufrusine *et al*. 2022 | Yes | Yes | NA | Yes | Yes | Yes | Yes | Yes |
| Hamrefors *et al*. 2024 | Yes | Yes | NA | Yes | Yes | Yes | Yes | Yes |
| Iosef *et al*. 2022 | Yes | Yes | NA | Yes | NA | NA | Yes | Yes |
| Kovarik *et al.* 2023 | Yes | Yes | NA | Yes | Yes | Yes | Yes | Yes |
| Kruger *et al*. 2022 | Yes | Yes | NA | Yes | Yes | Yes | Yes | Yes |
| Mahdi *et al*. 2024 | Yes | Yes | NA | Yes | Yes | NA | Yes | Yes |
| Peppercorn *et al*. 2023 | Yes | Yes | NA | Yes | Yes | No | Yes | Yes |
| Saito *et al*. 2024 | Yes | Yes | NA | Yes | Yes | No | Yes | Yes |
| Taenzer *et al*. 2023 | Yes | Yes | NA | Yes | Yes | No | Yes | Yes |
| Zhao *et al*. 2022 | Yes | Yes | NA | Yes | No | No | Yes | Yes |

**NA=Not applicable, ? = Unclear*

| 1. Were the criteria for inclusion in the sample clearly defined? |
| --- |
| 1. Were the study subjects and the setting described in detail? |
| 1. Was the exposure measured in a valid and reliable way? |
| 1. Were objective, standard criteria used for measurement of the condition? |
| 1. Were confounding factors identified? |
| 1. Were strategies to deal with confounding factors stated? |
| 1. Were the outcomes measured in a valid and reliable way? |
| 1. Was appropriate statistical analysis used? |
|  |

Table S2. Cohort studies

| Study | 1 | 2 | 3 | 4 | 5 | 6 | 7 | 8 | 9 | 10 | 11 |
| --- | --- | --- | --- | --- | --- | --- | --- | --- | --- | --- | --- |
| Brīvība *et al*. 2023 | Yes | NA | NA | Yes | Yes | NA | Yes | Yes | ? | No | Yes |
| Cervia-Hasler *et al*. 2024 | Yes | NA | NA | Yes | Yes | NA | Yes | Yes | NA | NA | Yes |
| García-Hildago *et al*. 2023 | Yes | NA | NA | Yes | Yes | NA | Yes | Yes | ? | ? | Yes |
| Greene *et al*. 2024 | Yes | NA | NA | Yes | Yes | NA | Yes | Yes | ? | ? | Yes |
| Liu *et al*. 2022 | Yes | NA | NA | Yes | Yes | NA | Yes | Yes | ? | ? | Yes |
| López Hernández *et al.* 2023 | Yes | NA | NA | Yes | Yes | NA | Yes | Yes | NA | NA | Yes |
| Ryan *et al*. 2022 | Yes | NA | NA | Yes | Yes | NA | Yes | Yes | ? | ? | Yes |
| Sanhueza *et al*. 2023 | Yes | NA | NA | Yes | Yes | NA | Yes | Yes | ? | ? | Yes |
| Sykes *et al*. 2023 | Yes | NA | NA | Yes | Yes | NA | Yes | NA | NA | NA | Yes |
| Vijayakumar *et al*. 2022 | Yes | NA | NA | Yes | Yes | NA | Yes | NA | NA | NA | Yes |
| Visvabarathy *et al*. 2022 | Yes | NA | NA | ? | ? | NA | Yes | ? | ? | ? | Yes |
| Wang *et al*. 2023 | Yes | NA | NA | Yes | Yes | NA | Yes | Yes | ? | ? | Yes |
| Zhang *et al*. 2023 | Yes | NA | NA | Yes | Yes | NA | Yes | Yes | ? | NA | Yes |

**NA=Not applicable, ? = Unclear*

| 1. Were the two groups similar and recruited from the same population? |
| --- |
| 1. Were the exposures measured similarly to assign people to both exposed and unexposed groups? |
| 1. Was the exposure measured in a valid and reliable way? |
| 1. Were confounding factors identified? |
| 1. Were strategies to deal with confounding factors stated? |
| 1. Were the groups/participants free of the outcome at the start of the study (or at the moment of exposure)? |
| 1. Were the outcomes measured in a valid and reliable way? |
| 1. Was the follow up time reported and sufficient to be long enough for outcomes to occur? |
| 1. Was follow up complete, and if not, were the reasons to loss to follow up described and explored? |
| 1. Were strategies to address incomplete follow up utilized? |
| 1. Was appropriate statistical analysis used? |
|  |
|  |

Table S3. Case-control studies

| Study | 1 | 2 | 3 | 4 | 5 | 6 | 7 | 8 | 9 | 10 |
| --- | --- | --- | --- | --- | --- | --- | --- | --- | --- | --- |
| Aschman *et al*. 2023 | Yes | Yes | Yes | NA | NA | Yes | Yes | Yes | NA | Yes |
| Guo *et al*. 2023 | Yes | Yes | Yes | NA | NA | Yes | ? | Yes | NA | Yes |
| Kazantseva *et al*. 2023 | Yes | Yes | Yes | NA | NA | No | No | Yes | NA | Yes |
| Medori *et al*. 2023 | Yes | Yes | Yes | NA | NA | Yes | ? | Yes | NA | Yes |
| Taylor *et al*. 2023 | Yes | Yes | Yes | NA | NA | Yes | Yes | Yes | NA | Yes |

**NA=Not applicable, ? = Unclear*

| 1. Were the groups comparable other than the presence of disease in cases or the absence of disease in controls? |
| --- |
| 1. Were cases and controls matched appropriately? |
| 1. Were the same criteria used for identification of cases and controls? |
| 1. Was exposure measured in a standard, valid and reliable way? |
| 1. Was exposure measured in the same way for cases and controls? |
| 1. Were confounding factors identified? |
| 1. Were strategies to deal with confounding factors stated? |
| 1. Were outcomes assessed in a standard, valid and reliable way for cases and controls? |
| 1. Was the exposure period of interest long enough to be meaningful? |
| 1. Was appropriate statistical analysis used? |

**Data extraction**

Table S4. Extracted data from included studies on time points of LC sample collection, outcome parameters and omics analysis platforms.

| **Study (lead author, year of publication)** | **Time points of LC sample collection** | **Outcome parameters** | **Omics analysis platforms** |
| --- | --- | --- | --- |
| **2021** | | | |
| Zhao *et al.*, 2021(1) | >3 months post-COVID | Protein expression profiling, specific IgG antibodies and neutralization rates | Olink proteomics proximity extension assay |
| **2022** | | | |
| Liu *et al.*, 2022(2) | 6 months post-discharge | Differences in gut microbiota composition | Shotgun metagenomic sequencing |
| Kruger *et al.*. 2022 (3) | Average of 221 ± 99 days post-COVID | Inflammatory and coagulation-related biomarkers, such as IL-6, von Willebrand factor and Platelet Factor 4. Self-reported symptoms, like fatigue, cognitive impairment, and dyspnea. | LC-MS |
| Ryan *et al.*, 2022(4) | 12, 16, 24 weeks post-COVID | Gene expression profiling, Immune cell populations, antibody responses | RNA-seq with Illumina 6000 NovaSeq |
| Vijayakumar *et al*., 2022(5) | 3–6 months post hospitalization | Protein expression profiling, immune cell composition, clinical outcomes (CT, lung function) | Olink proteomic proximity extension assay |
| **2023** | | | |
| Aschman *et al*., 2023(6) | 12 months post-COVID | Gene and protein expression profiles, capillary alterations, immune dysregulations, type-2b-fiber atrophy, complement and coagulation cascade related proteins | Illumina NovaSeq 6000 RNA-seq, proteomics using UltiMate 3000 RSLC nano UHPLC coupled to a  QExactive HF mass spectrometer |
| Berezhnoy, *et al*., 2023(7) | Median day of sample collection 152 days post-COVID | Metabome profiling, lipoprotein, and cytokine levels | H-NMR spectroscopy |
| Dufrusine, *et al*., 2023(8) | 3-6 months post-COVID | Protein expression profiling related to iron metabolism, 5-LOX protein levels and LTB4 products, cytokine levels | LC-MS/MS for proteomics |
| Guo *et al*., 2023(9) | 134–498 days post-COVID | Metabolome profiling, mitochondrial function, autophagy, apoptosis | UPLC-MS/MS |
| Iosef, *et al*., 2023(10) | 101.5 days post-COVID | Protein expression profiling, including protein biomarkers and signaling pathways related to vasculo-proliferative disease, immune cell redistribution, and organ-specific proteomic changes | Olink proteomics proximity extension assay |
| Kazantseva *et al.,* 2023(11) | During acute COVID-19 | Genetic profiling, neurological complications, anosmia, memory problems, sleep issues, depression | Illumina IScan |
| Kovarik *et al.,* 2023(12) | > 3 months post-SARS-CoV-2 infection or post-vaccination | Metabolome, proteome expression profiling, cytokine levels, fatty acid and oxylipin analysis | Untargeted shotgun proteomics, targeted metabolomics assay |
| López-Hernández *et al*., 2023(13) | >20 months post-COVID | Metabolome profiling including 108 metabolites | LC–MS/MS and FIA MS/MS for targeted metabolomics |
| Mahdi *et al.*, 2023(14) | 18 months post-COVID | Protein expression profiling, cytokines/chemokines, sphingolipid levels | Olink proteomics proximity extension assay |
| Medori *et al*., 2023(15) | >6 months post-COVID | Protein expression profiling, identification of inflammatory biomarkers and correlations with anxiety, fatigue, dyspnea and cognitive deficits | LC MS/MS |
| Peppercorn *et al*., 2023(16) | 12 months post-COVID | Protein expression profiling, with a focus on immune function and mitochondrial functions | Sequential Window Acquisition of all Theoretical Fragment Ion Spectra-Mass Spectrometry (SWATH-MS) |
| Sanhueza *et al*., 2023(17) | 4 months and 12 months post-COVID | Protein expression profiling, pulmonary function, CT scan, cytokine levels, metabolic syndrome parameters | nLC-MS/MS |
| Sykes *et al*., 2023(18) | 3 months post-COVID | Gene expression profiling, Enhanced vascular fibrosis, myosin light change phosphorylation, endothelium-independent and -dependent vasorelaxation, vasoconstriction | Spatial transcriptomics (Nanostring GeoMx Digital Spatial Profiler) |
| Taenzer *et al*., 2023(19) | >6 months post-COVID | Metabolite profiling, levels of neurotransmitter precursors phenylalanine, tryptophan and related downstream metabolites, associations with symptoms (anxiety, fatigue, depression) | LC-MS |
| Taylor *et al*., 2023(20) | During acute COVID-19 | Genetic variants associated with LC, specifically severe and fatigue-dominant phenotypes | Illumina Global Screening Array for genotypic data with Multi-disease drop-in panel |
| Visvabharathy *et al*., 2023(21) | 162.3-214.7 days post-symptom onset | Protein expression profiling, T cell and antibody responses to SARS-CoV-2 Nucleocapsid protein, cognitive function, quality of life | SOMAscan for proteomics |
| Wang *et al*., 2023(22) | 6 months post-COVID | Protein expression and metabolome profiling, changes in cytokine levels, health-related quality-of-life scores; clinical outcomes | Targeted plasma proteomics and metabolomics by LC-MS |
| Zhang *et al*., 2023(23) | 3 months post-COVID | Gut microbiota composition, oral microbiota diversity, metabolome profiling, correlation with gastrointestinal symptoms of LC | Shotgun metagenomic sequencing, 2bRAD-M sequencing, UPLC‒MS/MS for metabolomics |
| **2024** | | | |
| Brīvība *et al*., 2024(24) | 3 months post-COVID | Changes in the gut microbiome composition and diversity | DNBSEQ-G400RS sequencing platform |
| Cervia-Hasler *et al*., 2024(25) | 6 months post-COVID | Gene and protein expression profiling, Complement activation, thrombo-inflammation, tissue injury markers, endothelial and platelet activation, immune response markers | SomaScan for serum proteomics, scRNA-seq using Illumina NovaSeq 6000 |
| García-Hidalgo *et al*., 2024(26) | Short-term (median = 94 days post-discharge), Long-term (median = 358 days post-discharge) | Gene and protein expression profiling, pulmonary function | miRCURY LNA Universal RT microRNA PCR System |
| Greene *et al*., 2024(27) | At 6 and 12 months post-COVID-19 | Gene expression profiles, BBB (Blood-Brain Barrier) dysfunction, cognitive impairment | Illumina NovaSeq 6000 RNA-seq |
| Hamrefors *et al*., 2024(28) | >3 months post-COVID | Differences in gut microbiota composition, diversity, and functional abundances and correlations with fatigue and gastrointestinal symptoms | Illumina platform for DNA sequencing |
| Saito *et al*., 2024(29) | ~12 months post-COVID | Metabolome profiling, soluble biomarkers, pro-inflammatory cytokines, auto-antibodies | LC-MS |

*LC-MS= liquid chromatography-mass spectrometry, (sc)RNA-sequencing=(single-cell) RNA-sequencing, UPLC=ultra-performance liquid chromatography*

Table S5. Extracted data from included studies on SARS-CoV-2-specific antibody measurements and reinfection assessments.

| **Study (lead author, year of publication)** | **Measured SARS-CoV-2 IgG levels** | **Longitudinal SARS-CoV-2**  **IgG measurements** | **Reinfection assessment** | **SARS-CoV-2**  **specific igG target** |
| --- | --- | --- | --- | --- |
| **2021** | | | |  |
| Zhao *et al.*, 2021(1) | Yes | No | No | Anti-S |
| **2022** | | | |  |
| Liu *et al.*, 2022(2) | No | No | No | NA |
| Kruger *et al.*. 2022 (3) | No | No | No | NA |
| Ryan *et al.*, 2022(4) | Yes | Yes | No | Anti-RBD, anti-S |
| Vijayakumar *et al*., 2022(5) | Yes | No | No | Anti-RBD |
| **2023** | | | |  |
| Aschman *et al*., 2023(6) | Yes | No | No | Anti-N,  anti-S |
| Berezhnoy, *et al*., 2023(7) | No | No | No | NA |
| Dufrusine, *et al*., 2023(8) | No | No | No | NA |
| Guo *et al*., 2023(9) | No | No | No | NA |
| Iosef, *et al*., 2023(10) | No | No | No | NA |
| Kazantseva *et al.,* 2023(11) | No | No | No | NA |
| Kovarik *et al.,* 2023(12) | No | No | No | NA |
| López-Hernández *et al*., 2023(13) | No | No | No | NA |
| Mahdi *et al.*, 2023(14) | No | No | No | NA |
| Medori *et al*., 2023(15) | No | No | No | NA |
| Peppercorn *et al*., 2023(16) | No | No | No | NA |
| Sanhueza *et al*., 2023(17) | Yes | No | No | Anti-N, anti-S |
| Sykes *et al*., 2023(18) | No | No | No | NA |
| Taenzer *et al*., 2023(19) | No | No | No | NA |
| Taylor *et al*., 2023(20) | No | No | No | NA |
| Visvabharathy *et al*., 2023(21) | Yes | No | No | Anti-RBD |
| Wang *et al*., 2023(22) | No | No | No | NA |
| Zhang *et al*., 2023(23) | No | No | No | NA |
| **2024** | | | |  |
| Brīvība *et al*., 2024(24) | No | No | No | NA |
| Cervia-Hasler *et al*., 2024(25) | Yes | No | No | Anti-RBD, anti-S |
| García-Hidalgo *et al*., 2024(26) | No | No | No | NA |
| Greene *et al*., 2024(27) | No | No | No | NA |
| Hamrefors *et al*., 2024(28) | No | No | No | NA |
| Saito *et al*., 2024(29) | No | No | No | NA |

**NA=not applicable, N=nucleocapsid protein, RBD=receptor-binding domain, S=spike protein.***References**

1. Zhao J, Schank M, Wang L, Dang X, Cao D, Khanal S, et al. Plasma biomarkers for systemic inflammation in COVID-19 survivors. Proteomics Clin Appl. 2022 Sep;16(5):e2200031.

2. Liu Q, Mak JWY, Su Q, Yeoh YK, Lui GCY, Ng SSS, et al. Gut microbiota dynamics in a prospective cohort of patients with post-acute COVID-19 syndrome. Gut. 2022 Mar;71(3):544–52.

3. Kruger A, Vlok M, Turner S, Venter C, Laubscher GJ, Kell DB, et al. Proteomics of fibrin amyloid microclots in long COVID/post-acute sequelae of COVID-19 (PASC) shows many entrapped pro-inflammatory molecules that may also contribute to a failed fibrinolytic system. Cardiovasc Diabetol. 2022 Sep 21;21(1):190.

4. Ryan FJ, Hope CM, Masavuli MG, Lynn MA, Mekonnen ZA, Yeow AEL, et al. Long-term perturbation of the peripheral immune system months after SARS-CoV-2 infection. BMC Med. 2022 Jan 14;20(1):26.

5. Vijayakumar B, Boustani K, Ogger PP, Papadaki A, Tonkin J, Orton CM, et al. Immuno-proteomic profiling reveals aberrant immune cell regulation in the airways of individuals with ongoing post-COVID-19 respiratory disease. Immunity. 2022 Mar;55(3):542-556.e5.

6. Aschman T, Wyler E, Baum O, Hentschel A, Rust R, Legler F, et al. Post-COVID exercise intolerance is associated with capillary alterations and immune dysregulations in skeletal muscles. Acta Neuropathol Commun. 2023 Dec 8;11(1):193.

7. Berezhnoy G, Bissinger R, Liu A, Cannet C, Schäfer H, Kienzle K, et al. Maintained imbalance of triglycerides, apolipoproteins, energy metabolites and cytokines in long-term COVID-19 syndrome patients. Front Immunol. 2023;14:1144224.

8. Dufrusine B, Valentinuzzi S, Bibbò S, Damiani V, Lanuti P, Pieragostino D, et al. Iron Dyshomeostasis in COVID-19: Biomarkers Reveal a Functional Link to 5-Lipoxygenase Activation. Int J Mol Sci. 2022 Dec 20;24(1):15.

9. Guo L, Appelman B, Mooij-Kalverda K, Houtkooper RH, van Weeghel M, Vaz FM, et al. Prolonged indoleamine 2,3-dioxygenase-2 activity and associated cellular stress in post-acute sequelae of SARS-CoV-2 infection. EBioMedicine. 2023 Aug;94:104729.

10. Iosef C, Knauer MJ, Nicholson M, Van Nynatten LR, Cepinskas G, Draghici S, et al. Plasma proteome of Long-COVID patients indicates HIF-mediated vasculo-proliferative disease with impact on brain and heart function. J Transl Med. 2023 Jun 10;21(1):377.

11. Kazantseva A, Enikeeva R, Takhirova Z, Davydova Y, Mustafin R, Malykh S, et al. Host Genetic Variants Linked to COVID-19 Neurological Complications and Susceptibility in Young Adults-A Preliminary Analysis. J Pers Med. 2023 Jan 6;13(1).

12. Kovarik JJ, Bileck A, Hagn G, Meier-Menches SM, Frey T, Kaempf A, et al. A multi-omics based anti-inflammatory immune signature characterizes long COVID-19 syndrome. iScience. 2023 Jan 20;26(1):105717.

13. López-Hernández Y, Monárrez-Espino J, López DAG, Zheng J, Borrego JC, Torres-Calzada C, et al. The plasma metabolome of long COVID patients two years after infection. Sci Rep. 2023 Aug 1;13(1):12420.

14. Mahdi A, Zhao A, Fredengren E, Fedorowski A, Braunschweig F, Nygren-Bonnier M, et al. Dysregulations in hemostasis, metabolism, immune response, and angiogenesis in post-acute COVID-19 syndrome with and without postural orthostatic tachycardia syndrome: a multi-omic profiling study. Sci Rep. 2023;13(1):20230.

15. Medori MC, Dhuli K, Tezzele S, Micheletti C, Maltese PE, Cecchin S, et al. Serum proteomic profiling reveals potential inflammatory biomarkers in long-COVID patients: a comparative analysis with healthy controls. Eur Rev Med Pharmacol Sci. 2023 Dec;27(6 Suppl):1–12.

16. Peppercorn K, Edgar CD, Kleffmann T, Tate WP. A pilot study on the immune cell proteome of long COVID patients shows changes to physiological pathways similar to those in myalgic encephalomyelitis/chronic fatigue syndrome. Sci Rep. 2023 Dec 12;13(1):22068.

17. Sanhueza S, Vidal MA, Hernandez MA, Henriquez-Beltran ME, Cabrera C, Quiroga R, et al. Clinical and pulmonary function analysis in long-COVID revealed that long-term pulmonary dysfunction is associated with vascular inflammation pathways and metabolic syndrome. Front Med (Lausanne). 2023;10:1271863.

18. Sykes RA, Neves KB, Alves-Lopes R, Caputo I, Fallon K, Jamieson NB, et al. Vascular mechanisms of post-COVID-19 conditions: rho-kinase is a novel target for therapy. Eur Heart J Cardiovasc Pharmacother. 2023 Apr 5;pvad025.

19. Taenzer M, Löffler-Ragg J, Schroll A, Monfort-Lanzas P, Engl S, Weiss G, et al. Urine Metabolite Analysis to Identify Pathomechanisms of Long COVID: A Pilot Study. Int J Tryptophan Res. 2023;16:11786469231220781.

20. Taylor K, Pearson M, Das S, Sardell J, Chocian K, Gardner S. Genetic risk factors for severe and fatigue dominant long COVID and commonalities with ME/CFS identified by combinatorial analysis. J Transl Med. 2023 Nov 1;21(1):775.

21. Visvabharathy L, Hanson BA, Orban ZS, Lim PH, Palacio NM, Jimenez M, et al. Neuro-PASC is characterized by enhanced CD4+ and diminished CD8+ T cell responses to SARS-CoV-2 Nucleocapsid protein. Front Immunol. 2023;14:1155770.

22. Wang K, Khoramjoo M, Srinivasan K, Gordon PMK, Mandal R, Jackson D, et al. Sequential multi-omics analysis identifies clinical phenotypes and predictive biomarkers for long COVID. Cell Rep Med. 2023 Nov 21;4(11):101254.

23. Zhang D, Weng S, Xia C, Ren Y, Liu Z, Xu Y, et al. Gastrointestinal symptoms of long COVID-19 related to the ectopic colonization of specific bacteria that move between the upper and lower alimentary tract and alterations in serum metabolites. BMC Med. 2023 Jul 19;21(1):264.

24. Briviba M, Silamikele L, Birzniece L, Ansone L, Megnis K, Silamikelis I, et al. Gut Microbiome Composition and Dynamics in Hospitalized COVID-19 Patients and Patients with Post-Acute COVID-19 Syndrome. INTERNATIONAL JOURNAL OF MOLECULAR SCIENCES. 2024 Jan;25(1).

25. Cervia-Hasler C, Brüningk SC, Hoch T, Fan B, Muzio G, Thompson RC, et al. Persistent complement dysregulation with signs of thromboinflammation in active Long Covid. Sci. 2024;383(6680):1–18.

26. García-Hidalgo MC, Benítez ID, Perez-Pons M, Molinero M, Belmonte T, Rodríguez-Muñoz C, et al. MicroRNA-guided drug discovery for mitigating persistent pulmonary complications in critical COVID-19 survivors: A longitudinal pilot study. Br J Pharmacol. 2024 Feb 15;

27. Greene C, Connolly R, Brennan D, Laffan A, O’Keeffe E, Zaporojan L, et al. Blood-brain barrier disruption and sustained systemic inflammation in individuals with long COVID-associated cognitive impairment. Nat Neurosci. 2024 Feb 22;

28. Hamrefors V, Kahn F, Holmqvist M, Carlson K, Varjus R, Gudjonsson A, et al. Gut microbiota composition is altered in postural orthostatic tachycardia syndrome and post-acute COVID-19 syndrome. Sci Rep. 2024 Feb 9;14(1):3389.

29. Saito S, Shahbaz S, Luo X, Osman M, Redmond D, Cohen Tervaert JW, et al. Metabolomic and immune alterations in long COVID patients with chronic fatigue syndrome. Front Immunol. 2024;15:1341843.
